# Tissue transglutaminase in the pathogenesis of heart failure

**DOI:** 10.1038/s41418-017-0028-9

**Published:** 2017-12-13

**Authors:** Arti V. Shinde, Nikolaos G. Frangogiannis

**Affiliations:** The Wilf Family Cardiovascular Research Institute, Department of Medicine (Cardiology), Albert Einstein College of Medicine, Bronx, NY USA

**Keywords:** Biophysical chemistry, Cardiomyopathies

First described in the 1950s, tissue tranglutaminase (tTG, transglutaminase 2), is a unique multifunctional member of the transglutaminase family with effects that extend beyond its enzymatic actions. A recently published study demonstrates an important role for endogenous tTG in the pathogenesis of pressure overload-induced heart failure, suggesting crosslinking actions that promote diastolic dysfunction, and protective matrix-preserving effects that prevent chamber dilation [1]. The functions of tTG in the failing heart may involve both enzymatic and non-enzymatic effects with several different cellular targets and a wide range of molecular interactions.

Ca2+-dependent transamidation, the best characterized enzymatic function of tTG, modifies proteins by crosslinking their reactive carboxamide side chains to primary amines [2]. tTG also exerts enzymatic functions that do not require Ca2+, acting as a GTPase, protein kinase or protein disulfide isomerase, and participates in non-enzymatic interactions with many different proteins, serving adapter and signaling functions both within and outside the cells [3]. In vitro studies have implicated tTG in a wide range of cellular functions, including cell survival, adhesion and migration, cell growth, proliferation, and differentiation [4]. The absence of significant baseline phenotypic abnormalities in mice with global loss of tTG [5] demonstrated that, despite its broad repertoire of presumed cellular functions, tTG does not play a crucial role in maintaining tissue homeostasis. In contrast, a growing body of evidence suggests that tTG is overexpressed and activated following tissue injury, and may regulate pathophysiologic responses.

## tTG in heart disease

tTG is constitutively expressed in normal mammalian myocardium, and is upregulated in injured and failing hearts [6]. In a recently published study [1], we provided the first evidence suggesting an important role for tissue transglutaminase in the pathogenesis of heart failure. We used a mouse model of transverse aortic constriction that recapitulates the cardiomyopathy of pressure overload, one of the most common pathophysiologic conditions associated with heart failure in human patients. In the pressure-overloaded heart, early development of cardiac hypertrophy and fibrosis increases cardiac stiffness causing diastolic ventricular dysfunction, despite preserved systolic function. At a later stage, persistent pressure overload leads to progressive ventricular dilation, decompensation, and development of systolic heart failure. tTG was the only member of the transglutaminase family that is upregulated in the pressure-overloaded mouse myocardium. In remodeling hearts, tTG was localized in cardiomyocytes, macrophages, and interstitial cells, and was deposited in the extracellular matrix. Experiments in tTG knockout mice suggested that endogenous tTG plays no significant role in cardiac homeostasis, but participates in cardiac remodeling following pressure overload, exerting both detrimental and protective actions. tTG promoted collagen crosslinking, increasing chamber stiffness following pressure overload, but also protected the remodeling heart from dilation, preventing excessive matrix metalloproteinase activation, and restraining fibroblast proliferation. These observations highlight the functional complexity of tTG, but also generate several important questions:

## Which stimuli induce tTG expression and secretion in the remodeling myocardium?

Cardiac pressure overload activates mechanosensitive signaling pathways in all myocardial cells. In response to biomechanical stress, cardiomyocytes activate integrins, transducing downstream mitogen-activated protein kinase cascades, that may be involved in induction of tTG transcription [7]. Mechanosensitive signaling may also stimulate tTG transcription by locally activating transforming growth factor (TGF)-β through integrin-dependent actions [8], and through induction of TGF-β-activating matricellular proteins. Our findings suggested that TGF-β upregulates tTG expression in both cardiac fibroblasts and macrophages through distinct signaling pathways and may play an important role in tTG induction in vivo.

## What are the main cellular targets of tTG in the failing heart?

**Fig. 1:**
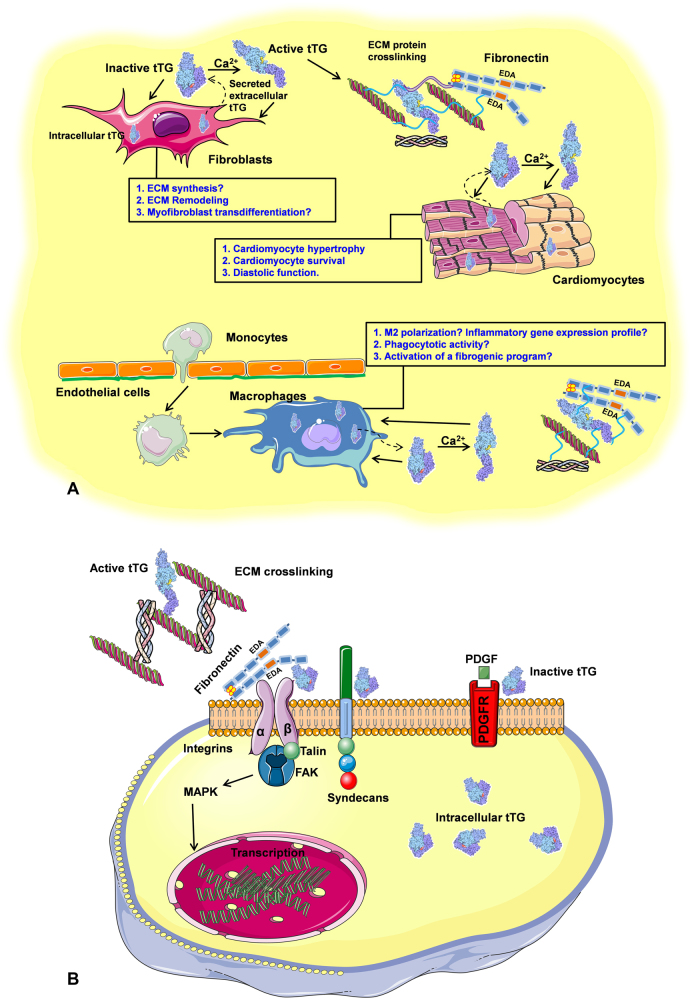
The cellular and molecular targets of tTG in the failing heart. ** a** In the pressure-overloaded myocardium, tTG is upregulated and secreted in the interstitium. Activated tTG plays a role in extracellular matrix (ECM) crosslinking, promoting diastolic dysfunction. Both enzymatically active and inactive tTG may exert effects on cardiomyocytes, fibroblasts, and macrophages by regulating intracellular signaling cascades, or by modulating outside-in signaling. In fibroblasts, tTG may modulate ECM synthesis and remodeling and may play role in myofibroblast activation. In cardiomyocytes, tTG may promote hypertrophic response, may modulate cell survival in response to stress and may play a role in diastolic dysfunction. Polarized M2 macrophages express tTG. tTG may be implicated in phagocytotic function of macrophages and may play a role in regulating inflammatory gene synthesis and macrophage-driven fibroblast activation. **b** tTG may interact with a wide range of proteins in the cell surface, cytoplasm, or ECM. Active tTG may promote ECM crosslinking. tTG may also modulate signaling responses in a transamidase-independent manner. tTG may facilitate fibronectin-activated integrin signaling transducing a focal adhesion kinase (FAK) cascade, may bridge integrins with growth factor receptors (such as platelet-derived growth factor receptor/PDGFR), and may activate syndecan-mediated responses. Additional symbol: MAPK, mitogen-activated protein kinase

Considering its ubiquitous expression, its secretion and deposition in the activated extracellular matrix, and its broad functional repertoire, tTG may have a wide range of cellular targets in heart failure (Fig. 1). Published studies suggest important (and sometimes conflicting) effects of tTG on all cell types involved in cardiac remodeling. In cardiomyocytes, tTG overexpression activated cycloxygenase-2 signaling promoting cell death and inducing ventricular dysfunction [9]. In contrast, loss-of-function experiments suggested that tTG may protect cardiomyocytes from ischemic death [10]. tTG expression in fibroblasts promotes cell adhesion [11], regulates fibroblast migration, stimulates fibronectin synthesis and has been implicated in organization of the fibroblast-derived extracellular matrix network [12]. tTG is also expressed in macrophages, and has been implicated in recognition and phagocytosis of apoptotic cells by professional phagocytes [13]. A recent study demonstrated that tTG is a consistent and preserved marker of polarized M2 macrophages in humans and mice [14]; however, whether tTG regulates macrophage polarization in vivo remains unknown.

In our study, use of a mouse line with global loss of tTG precludes conclusions regarding the in vivo role of specific cellular responses in mediating the effects of tTG loss. Thus, our experiments did not explain the cellular basis for the strikingly increased mortality in tTG null mice undergoing pressure overload protocols. Accentuated dilative remodeling due to loss of protective matrix-preserving effects may be in part responsible for increased mortality in pressure-overloaded tTG knockout animals; however, the severity of systolic dysfunction could not account for the observed effect on survival. Whether endogenous tTG protects the myocardium from arrhythmogenesis or defective impulse conduction is unknown.

On the other hand, the observed effects on matrix remodeling and fibrosis may reflect important actions of tTG on fibroblasts, or effects on the fibrogenic phenotype of immune cells (such as macrophages). Our in vitro findings suggested that tTG restrains fibroblast proliferative capacity and promotes a matrix-preserving program in cardiac fibroblasts by inducing tissue inhibitor of metalloproteinases (TIMP)1 synthesis.

## Do the in vivo effects of tTG involve enzymatic effects

Although tTG expression is markedly induced in the remodeling myocardium, whether increased protein levels are associated with accentuated enzymatic activity remains unclear. In normal tissues, tTG activity is tightly regulated. In the cytoplasm, low Ca2+ concentrations prevent intracellular transamidation activity, and GTP/GDP act as allosteric inhibitors, reducing accessibility of the catalytic domain, and preventing inappropriate intracellular tTG activation. In the pressure-overloaded heart, increased calcium concentrations may co-operate with pro-inflammatory cytokines, triggering intracellular tTG activation. In the cardiac interstitium, high levels of Ca2+ may stimulate tTG-mediated transamidase activity, promoting crosslinking of extracellular matrix proteins and leading to formation of a protease-resistant matrix. The crosslinking actions of tTG may also serve to localize and activate TGF-β, further accentuating fibrosis. However, interstitial activation of tTG may be transient due to release of mediators that inactivate tTG, such as reactive oxygen and nitric oxide [15]. Thus, the relative significance of enzymatic tTG actions in the pathogenesis of cardiac remodeling is not known.

## What is the in vivo significance of non-enzymatic functions of tTG?

Non-enzymatic functions of tTG may play an important role in activation of cardiac interstitial cells and in the pathogenesis of myocardial fibrosis. Through binding with both fibronectin and heparin sulfate proteoglycans, tTG may function as a molecular bridge between the matrix and the cells, activating syndecan-4-mediated signaling [16]. tTG may also co-operate with fibronectin to trigger integrin activation and downstream stimulation of Focal adhesion kinase signaling; these effects are transamidase-independent [12]. Moreover, cell surface tTG may bridge β1 integrins with growth factor receptors [17]. In our study, matrix-bound tTG exerted matricellular effects on fibroblasts inducing TIMP1 expression in a transamidase-independent manner. The matricellular actions of tTG may be particularly important in vivo as they do not require persistent enzymatic activation.

## Future directions

Our recently published findings suggest that tTG is a critical stress-induced signal in the failing heart, exerting both profibrotic effects that increase ventricular stiffness and protective actions that preserve extracellular matrix integrity, preventing chamber dilation. Considering the potential translational significance of tTG-mediated actions in heart failure, study of the molecular basis for the effects of tTG is an important priority. Future experiments should focus on several different directions. First, dissection of the enzymatic and non-enzymatic actions of tTG in the remodeling myocardium and identification of the main substrates is important in order to design safe and effective therapeutic strategies. Second, investigation of the cellular targets of tTG in the remodeling heart is needed in order to identify specific cellular responses that could be targeted in human patients. Third, characterization of the molecular signals interacting with tTG and understanding of their role in mediating specific cellular responses can provide critical information on specific mechanisms of action with direct translational implications. Fourth, systematic analysis of changes in myocardial tTG expression in human failing hearts may provide insights into the role of tTG in specific heart failure subpopulations. Considering the heterogeneity of human heart failure, identification of patients with prominent fibrosis and excessive activation of myocardial tTG-mediated matrix crosslinking may mark a subpopulation that may benefit from attenuation of enzymatic tTG actions.

## References

[1] Shinde AV (2017). Cardiovasc Res.

[2] Kiraly R (2011). FEBS J.

[3] Eckert RL (2014). Physiol Rev.

[4] Nurminskaya MV (2012). Int Rev Cell Mol Biol.

[5] De Laurenzi V (2001). Mol Cell Biol.

[6] Petrak J (2011). Proteome Sci.

[7] Akimov SS (2003). J Biol Chem.

[8] van Putten S (2016). J Mol Cell Cardiol.

[9] Zhang Z (2003). Circ Res.

[10] Szondy Z (2006). Cell Death Differ.

[11] Gentile V (1992). J Cell Biol.

[12] Akimov SS (2001). Blood.

[13] Szondy Z (2003). Proc Natl Acad Sci USA.

[14] Martinez FO (2013). Blood.

[15] Lai TS (2001). Biochemistry.

[16] Telci D (2008). J Biol Chem.

[17] Zemskov EA (2009). J Biol Chem.

